# Lignin Nanoparticles and Alginate Gel Beads: Preparation, Characterization and Removal of Methylene Blue

**DOI:** 10.3390/nano12010176

**Published:** 2022-01-05

**Authors:** Tong Luo, Yanping Hao, Chao Wang, Weikun Jiang, Xingxiang Ji, Guihua Yang, Jiachuan Chen, Srinivas Janaswamy, Gaojin Lyu

**Affiliations:** 1 State Key Laboratory of Biobased Material and Green Papermaking, Qilu University of Technology, Shandong Academy of Sciences, Jinan 250353, China; luotongiant@gmail.com (T.L.); yanpingh2021@gmail.com (Y.H.); weikun0709@126.com (W.J.); jxx@qlu.edu.cn (X.J.); ygh@qlu.edu.cn (G.Y.); chenjc@qlu.edu.cn (J.C.); 2 Department of Dairy and Food Science, South Dakota State University, Brookings, SD 57007, USA

**Keywords:** lignin nanoparticles, deep eutectic solvents, sodium alginate, methylene blue, adsorption kinetics

## Abstract

A novel and effective green system consisting of deep eutectic solvent (DES) was proposed to prepare lignin nanoparticles (LNPs) without any lignin modification. The LNPs are obtained through the dialysis of the kraft lignin-DES solution. The particle size distribution, Zeta potential and morphology of the LNPs are characterized by using dynamic light scattering (DLS), scanning electron microscopy (SEM) and transmission electron microscopy (TEM). The average diameter of LNPs is in the range 123.6 to 140.7 nm, and the LNPs show good stability and dispersibility in water. The composite beads composed of LNPs and sodium alginate (SA) are highly efficient (97.1%) at removing methylene blue (MB) from the aqueous solution compared to 82.9% and 77.4% by the SA/bulk kraft lignin composite and pure SA, respectively. Overall, the LNPs-SA bio-nanocomposite with high adsorption capacity (258.5 mg/g) could be useful in improving water quality and other related applications.

## 1. Introduction

Lignins are natural polymers with aromatic chemical structure possessing methoxy, aldehyde, keto, hydroxyl and phenolic groups [1,2]. They are being extracted from a variety of sources, and large-scale production predominantly arises from the bioethanol and xylose/furfural industries [3,4]. Ironically, around 98% of lignin from the paper and/or pulp industries is being burned, considering it a waste by-product and non-optimized energy resource [5,6]. However, a plethora of applications could be envisioned from lignins, for example, in the synthesis of polymers, dyes and fertilizers, as well as as adhesives in ecological and low-carbon plywood [7]. Their value addition further expands as a green and sustainable source for the design and development of bio-adsorbents, conductive elastomers and packaging membranes [8]. In this regard, the effective valorization of lignin is of great interest, which is further fueled by the shortage of petrochemical resources.

The nonuniform and highly complex chemical structures of lignins also limit their wholesome use. Toward this, tailoring lignin’s size to submicron and/or nano scale could be one viable option [9,10]. Such a process, indeed, aids to overcome the non-homogeneity of lignin. More importantly, the resulting nano-character coupled with amounts of functional groups, large specific surface area, high diffusibility and compatibility, and low-cost nature opens up novel opportunities for lignins [11,12]. For example, lignin nanoparticles (LNPs) are helpful to fabricate novel and well-performing bio-nanocomposite materials [13,14,15,16,17,18]. Anti-ultraviolet (UV)/anti-bacterial coatings [19,20], the controlled release of drugs [21,22] and the adsorption of heavy metal ions [23] are a couple of proven examples.

To date, several protocols, such as sonication, pH precipitation, chemical cross-linking/polymerization, mechanical treatment and dialysis, on the preparation of LNPs have been developed [24,25,26,27]. However, most of these methods demand the chemical modification of lignin using toxic chemicals (e.g., acetyl bromide, pyridine) [12]. The size and shape of the thus prepared LNPs are irregular and aggregate during preparation [17]. In this set, however, dialysis gained special attention due to its simple operation, cost-effectiveness and ability to yield spherical LNPs [28]. During this process, lignin is being dissolved in organic solvents such as acetone, dioxane, dimethyl sulfoxide (DMSO) and tetrahydrofuran (THF) [29,30,31,32]. However, these are toxic, volatile, flammable and difficult to handle [33]. In addition, dialysis requires the use of larger quantities of solvents. In this regard, a simple method based on green solvents along with environmental friendliness and easy handling is highly desirable, and deep eutectic solvents (DES), formed through the combination of hydrogen bond acceptors (HBA) and hydrogen bond donors (HBD), stand out as viable alternatives. In recent years, DES gained considerable attention as a sustainable solution with high potential to dissolve and valorize lignin [34,35,36,37,38,39] but with little-to-no exploration of industrial lignins.

Herein, a green and effective approach for LNPs preparation using a novel alkaline DES system without lignin modification has been reported. The structure and morphology of LNPs were investigated using dynamic light scattering (DLS), scanning electron microscopy (SEM) and transmission electron microscopy (TEM). Furthermore, to realize the high-value utilization of LNPs, gel beads have been prepared by combining LNPs with sodium alginate (SA), and their propensity to capture methylene blue (MB) has been demonstrated. Sodium alginate (SA) is an inexpensive natural and abundant polysaccharide. Its non-toxicity, physiological inertness, biocompatibility and biodegradability gained it widespread utility in food and non-food applications [40,41]. The effect of LNPs content, contact time, temperature, dosage of adsorbent and initial MB concentration on the adsorption have been investigated, along with the adsorption efficiency and release kinetics.

## 2. Materials and Methods

### 2.1. Materials

**Figure 1:**
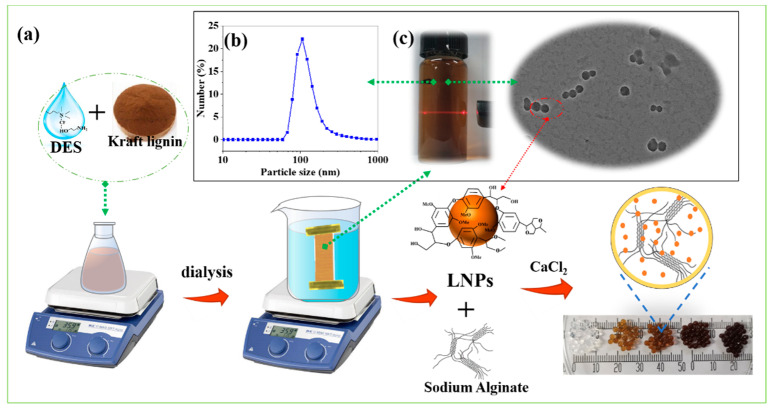
(**a**) Schematic representation of the preparation procedure for LNPs and SA/LNPs beads; (**b**) particle-size distribution. (**c**) The digital photo of the Tyndall effect of the LNPs suspension.

Kraft lignin (KL) was gift from Huatai Paper Co., Ltd. (Rizhao, China). The chemical composition of lignin is shown in Figure 1 and Table S1. Choline chloride (ChCl), ethanolamine (ETA), sodium alginate (SA), calcium chloride (CaCl_2_) and methylene blue (MB) were purchased from Aladdin Biochemical Technology Co., Ltd. (Shanghai, China). All these reagents were of analytical pure grade and used directly without further processing. Information related to MB dye is shown in Table S2.

DES was prepared according to our previous protocol [42]. Briefly, ChCl and ETA were used as HBA and HBD, respectively. ChCl and ETA, at a molar ratio of 1:6, were added in a glass vial and heated at 60 °C with constant stirring to obtain the clear liquid of DES.

### 2.2. Preparation of LNPs

LNPs were prepared using the solvent-antisolvent displacement method (dialysis). Briefly, 1.0 g kraft lignin was dissolved in 20 g DES with magnetic stirring (600 rpm) for 2 h at the ambient temperature. Subsequently, the lignin–DES solution was dialyzed (*M_w_* = 2000 of dialysis bag) in the deionized water for 72 h, with the periodic replacement of fresh water, to completely remove the DES. After adjusting the concentration (0.1–0.4 wt%), the LNPs dispersion was stored in a refrigerator (4 °C) for further use. The LNPs powder was obtained after rotary evaporation and freeze-drying.

### 2.3. Characterization of LNPs

Transmission electron microscopy (TEM) images were recorded using the JEM2100F TEM (JEM, Japan) with an acceleration voltage of 200 kV. Scanning electron microscopy (SEM) observations were carried out on a Regulus 8220 (Hitachi, Japan) at 5 kV. The Zeta potential as well as the average particle size of LNPs was investigated with the dynamic light scatterometer from Nano-ZS90, Malvern, UK.

### 2.4. Preparation of SA/LNPs Composite Bead

Composite gel beads were prepared by mixing SA and LNPs (Figure 1 and Figure S1). A certain amount of SA powder was added into different LNPs dispersions to get a concentration of 1 wt% mixture under constant stirring at ambient temperature for 3 h. Subsequently, the mixture was added dropwise to the CaCl_2_ solution (0.1 M). The beads were formed immediately and cured for 12 h. After washing with deionized water and removing the excess Ca^2+^, the SA/LNPs beads were used for adsorption experiments. A few were also freeze-dried for further characterization. The details about the SA/LNPs gel beads and corresponding chemical composition are listed in Table 1. The samples were coded as SA/LNP-xx, for brevity. For example, SA/LNP-40 represent beads with LNPs content of 40 wt.%. The SA/L-40 beads served as the control group that were prepared by mixing kraft lignin with sodium alginate.

### 2.5. Characterization of SA/LNPs Composite Beads

Surface morphology of the SA/LNPs gel beads was investigated with the SEM Regulus 8220 from Hitachi, Japan. The chemical compositions of the gel beads were assessed by FT-IR spectroscopy from Bruker, ALPHA, Germany in the scanning range of 4000–500 cm^−1^ at a resolution of 4 cm^−1^. Thermogravimetric analysis (TGA) of the SA powder and gel beads was carried out with the Q50 thermogravimetric analyzer from TA Instruments, New Castle, DE, USA.

### 2.6. Removal Efficiency of the Composite Gel Beads

A predetermined amount of gel beads was dispersed in the MB aqueous solution of 50 mL. The mixture was then incubated in an air bath constant temperature shaker with constant stirring for a given time. The MB concentrations was detected by the UV–visible spectrophotometer UV-2600 from Shimadzu, Japan, at 664 nm [43,44]. The corresponding adsorption capacity (*q_e_*, mg/g), adsorption capacity at time *t* (*q_t_*, mg/g) and the removal rate (*φ*, %) were calculated using the following three equations.

(1) $$q_{e}=\frac{\left(C_{0}-C_{e}\right)V}{m}$$

(2) $$q_{t}=\frac{\left(C_{0}-C_{t}\right)V}{m}$$

(3) $$\varphi =\frac{C_{0}-C_{e}}{C_{0}}\times 100\%$$

Herein, *C*_0_ (mg/L) represents the initial concentration of dye. The *Ce* and *C_t_* (mg/L) refer the concentration at adsorption equilibrium and at adsorption time *t*, respectively. The *V* (measured in *L*) is the volume of the solution and m (measured in g) the mass of the composite beads.

### 2.7. Kinetic Modelling and Isotherms

Four models namely Lagergren’s pseudo-first-order, pseudo-second-order and intraparticle diffusion were used to describe the adsorption process. The corresponding equations Equations (S1)–(S3), respectively, are listed in the support material. The adsorption isotherms are represented by the Langmuir, Freundlich and Temkin isotherm equations (Equations (S4)–(S6), respectively). The fundamental properties of Langmuir isotherm could be illustrated by a dimensionless constant *R_L_*, known as separation factor, with the Equation (S7).

## 3. Results and Discussion

### 3.1. LNPs Prepared by Dialysis in a ChCl & ETA System

Lignin contains both hydrophobic units and hydrophilic functional groups, and thus it is generally considered as an amphiphilic biopolymer [45]. This structure makes it possible for lignin to form uniform hydrophilic nano-micelles in aqueous solutions [46]. DES has been proven to solubilize lignin effectively [47,48,49], and ChCl and ETA are the most promising candidates for industrial applications because of their low viscosity, environmentally friendly and biodegradable nature. In this regard, herein we propose DES composed of ChCl and ETA as a novel dissolution system for the LNPs preparation. The unmodified kraft lignin was first dissolved in DES, and then subjected to dialysis in plenty of fresh water at the ambient temperature. During this process, lignin solubility decreases but with the LNPs formation. The forces that contribute to LNPs development include hydrophobic, hydrogen bonding and π-interactions along with van der Waals forces [10,50]. The resulting LNPs suspension is a uniform solution that readily displays the Tyndall effect (Figure 1c).

**Figure 2:**
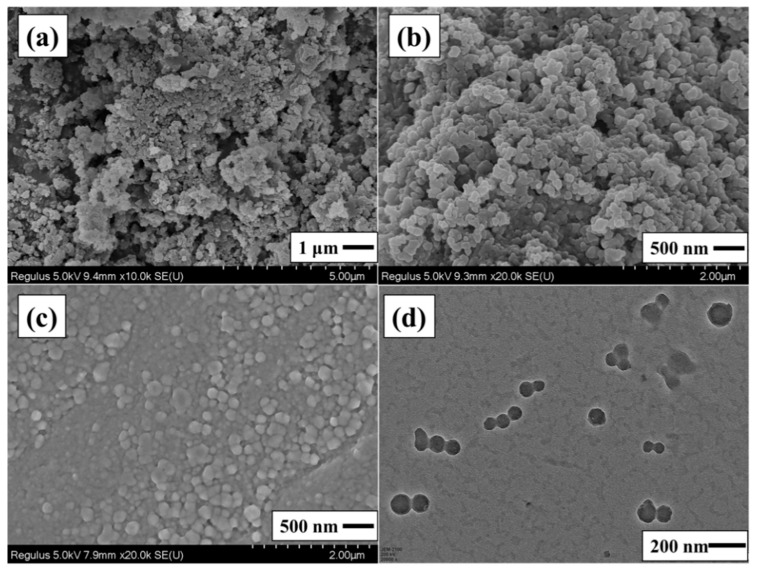
SEM images of the (**a**) lignin, (**b**) freeze-dried LNPs, (**c**) LNPs dispersions and (**d**) TEM images of the LNPs dispersions.

The mean particle diameter and size distribution of the LNPs are analyzed via a particle-size-versus-fraction distribution plot. The LNPs have a size of 60–200 nm (Figure 1b). The concentration of LNPs suspension did not affect the particle size, and the average is in the range of 123.6 to 140.7 nm for the 0.1 to 0.4% concentration (Figure S2). The SEM and TEM images are shown in Figure 2c,d, respectively. The initial kraft lignin particles possess an irregular shape with a micron size, but the prepared LNPs have more of a spherical morphology with a narrow nano-size distribution that was maintained even after freeze-drying (Figure 2). It can be seen that there are particle-size differences between the SEM/TEM and DLS studies. It may be because, in the process of dialysis, LNPs formed gradually in the aqueous medium with a hydrophilic surface and maintained the electrostatic stability with the presence of weak carboxyl groups of lignin that preclude particle aggregation, resulting in particles of average size. However, small amounts of LNPs could accumulate during the drying process of micromorphology analysis, resulting in a subtle increase in the particle size [17,32].

**Figure 3:**
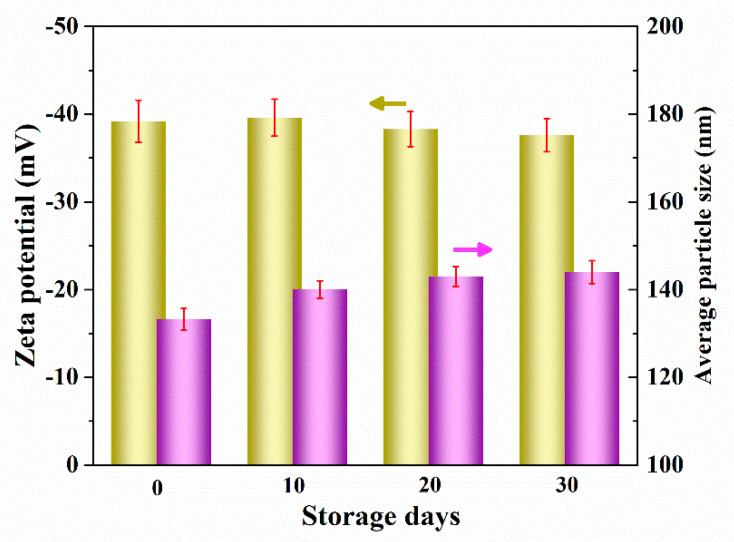
Average particle size and Zeta potential of the LNPs dispersions stored for 30 days.

The stability of LNPs is an important factor that influences their performance [51]. In order to evaluate LNPs, Zeta potential and average diameter are measured as a function of time. Interestingly, Zeta potential and the average diameter of LNPs change slightly (Figure 3) from −39.2 mV to −37.6 mV and from 133.2 nm to 144 nm during 30 days storage, respectively, portraying their stability in water. The high Zeta potential value clearly suggests that the ionizing groups and hydrophilic groups of LNPs are exposed on the nanoparticle surface, and the resulting strong repulsive forces prevent the LNPs aggregation, which certainly is advantageous [52,53]. The observed stability further hints that these LNPs could be explored as additives and stabilizers [54]. Overall, the regular spherical morphology, uniform particle size and high stability of these LNPs make them as promising candidates for the production of polymer nanocomposites but warrants further study.

### 3.2. Characterization of the Composite Gel Beads

**Figure 4:**
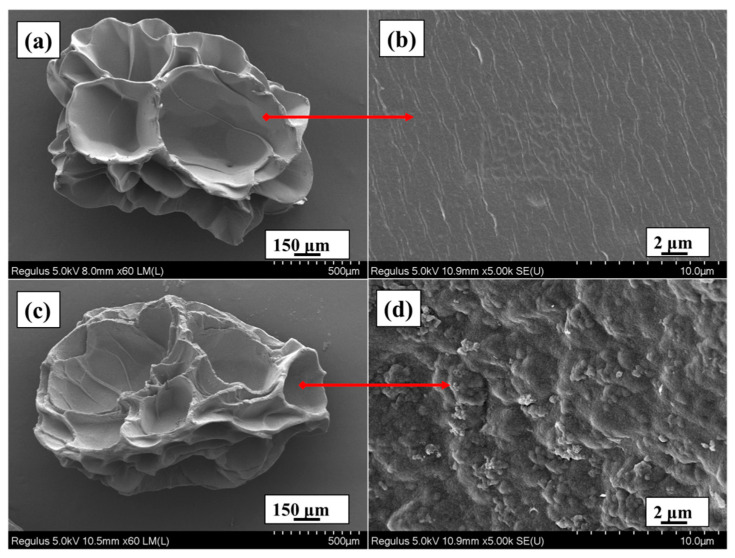
SEM images of the freeze-dried (**a**,**b**) SA beads and (**c**,**d**) SA/LNPs-40.

**Figure 5:**
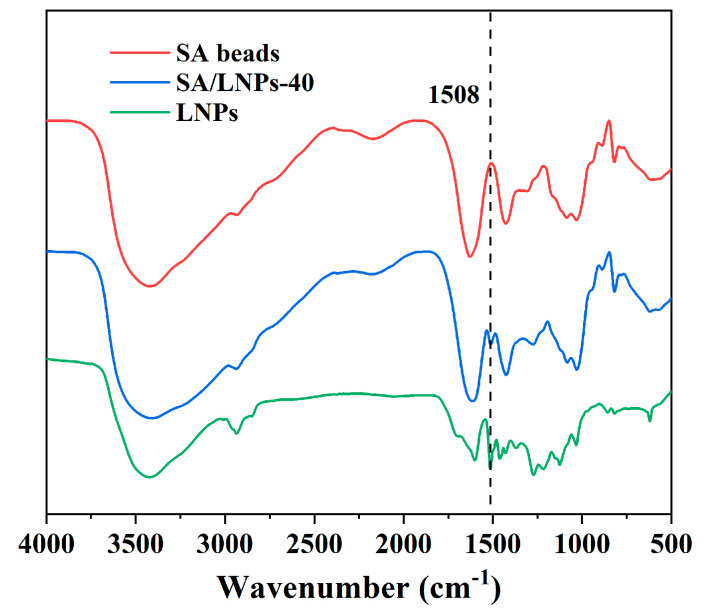
Comparison of the FT-IR spectra of LNPs, SA and SA/LNPs-40 beads.

The SA/LNPs composite beads are prepared by using a simple blending protocol. The addition of the LNPs/SA mixture into the Ca^2+^ solution improves the stability of the colloids (Figure S1). Thus, the introduction of LNPs into the alginate beads could improve their adsorption and thermal performance as well. The SEM images of the freeze-dried beads are shown in Figure 4. The SA beads display a smooth surface, but the incorporation of LNPs results in a corrugated surface (Figure 4d). Figure 5 highlights the FT-IR spectra of LNPs, SA beads and SA/LNPs-40 beads. In the LNPs, characteristic peaks at 1460 cm^−1^ correspond to the vibration of C−H deformation of lignin [55]. The peaks at 1508 cm^−1^ and 1024 cm^−1^ are due to C−C aromatic skeletal vibration and C−O stretching, respectively [56,57]. Compared with the pure SA beads, a new peak at 1508 cm^−1^ (C-C aromatic skeleton) in the SA/LNPs-40 beads suggest LNPs successful incorporation within the SA matrix.

**Figure 6:**
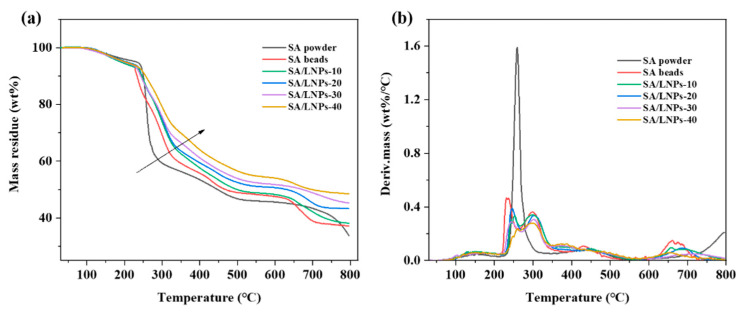
(**a**) TGA and (**b**) DTG curves of SA powder and composite beads.

The TG and DTG measurements are used to evaluate the thermal stability of the SA-LNPs composite beads. The thermal curves of SA powder, pure SA beads and composites beads with varying LNPs amount are shown in Figure 6. The initial degradation (*T_i_*) and maximum degradation (*T_max_*) temperature, along with the parameters of thermal stability, are compared in Table S3. The SA powder exhibits single-step degradation with a high *T_max_* of 259.3 °C, and the maximum rate reached is 1.59 wt%/°C. In contrast, composite beads exhibit relatively lower thermal decomposition temperature but with two degradation peaks, suggesting that the pyrolysis of composite beads could be divided into two steps. As the content of LNPs increases from 0 to 40%, the *T_i_* and *T_max_* raise from 225.3 to 239.2 °C and from 233.7 to 258.6 °C, respectively, but the maximum degradation rate decreases from 0.47 to 0.23 wt%/°C. The SA powder displays the least residue, of 33.7%, which increases with the addition of LNPs to 37.1–48.5% (Table S3). Overall, the thermal stability of SA beads improves with the incorporation of LNPs, presumably due to the formation of a cross-linked and/or condensed aromatic matrix [58].

### 3.3. Important Factors Affecting Adsorption

In recent years, biopolymers derived from biomass and natural materials have attracted research interest due to their low cost, ease of preparation and effective wastewater-treatment attributes [59,60]. In particular, their intrinsic ability to cleanup wastewater dyes that are toxic and carcinogenic has become an important environmental tool. Herein, the main mechanism for MB (cationic dyes) adsorption would be due to electrostatic interactions between the MB’s cationic groups and the carboxyl groups of the polysaccharide and the functional groups of lignin. The formation of hydrogen bonding and π-interactions could not be discounted, however [61]. The suggested mechanism of interactions between the dye and beads is highlighted in Figure S3. As it is well known that the adsorption efficiency depends on the physical and chemical properties of the adsorbent and dye, as well as the adsorption conditions such as time, temperature, dye and adsorbents concentration, etc. [62], the optimization of these parameters has been carried out to best accomplish the removal of MB by the SA-LNP beads.

#### 3.3.1. Effect of LNPs Content

**Figure 7:**
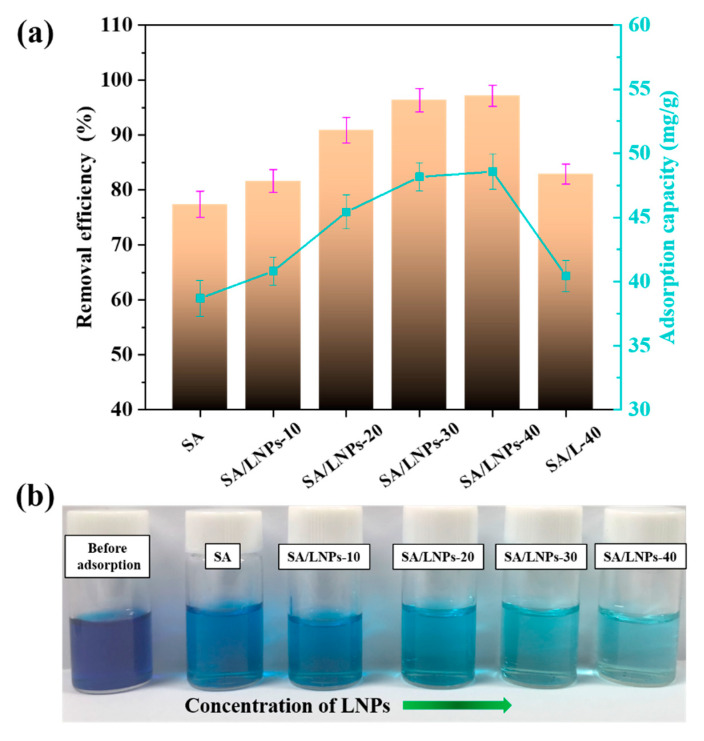
(**a**) Removal efficiency and adsorption capacity of MB by the SA-LNP composite beads with different LNPs content (2.0 g/L beads, 50 mL 100 mg/L MB, 150 rpm, 25 °C, 2 h), (**b**) Digital images of methylene blue absorbed by the SA-LNP beads with LNPs amount.

Interestingly, the proportion of LNPs could reach 40% without using any crosslinking agent. Further increase reduces the viscosity of the mixture and precludes bead formation in the calcium chloride solution. As seen from Figure 7b, it is clear that, with increase in the LNP content, the color of the MB solution gradually turns pale upon adsorption. This hints that the mass ratio of LNPs and SA had a great effect on the adsorption performance by the composite beads. The removal efficiency and adsorption capacity of the composite beads with different proportions for MB are shown in Figure 7a. The adsorption amount increases gradually with the LNPs amount. The equilibrium removal rate of SA/LNPs-40 beads reaches to 97.1% compared to 82.9% and 77.4% of SA/L and SA, respectively. Such an increase could be ascribed to the presence of LNPs in the beads [12].

#### 3.3.2. Effect of Contact Time

**Figure 8:**
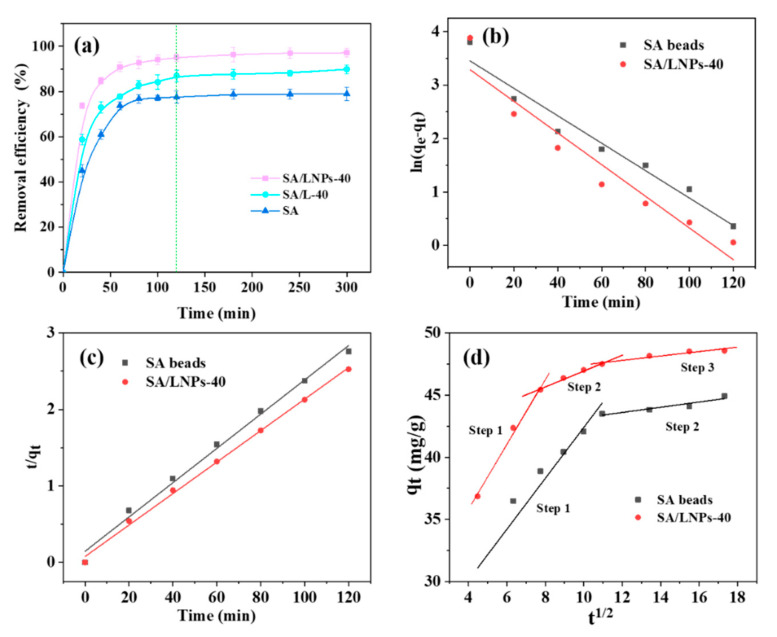
(**a**) The removal efficiency of different adsorption times (2.0 g/L beads, 50 mL 100 mg/L MB, 150 rpm, 25 °C); adsorption kinetics, linear fitting of (**b**) pseudo-first- and (**c**) pseudo-second-order kinetic models of SA and SA/LNPs-40 beads and (**d**) the intra-particle diffusion model of SA/LNPs-40 beads.

The adsorption capacity increases rapidly with time and later slows down to reach equilibrium at about 120 min (Figure 8a). Herein, Lagergren’s pseudo-first and second-order, as well as the intraparticle diffusion models, are tested to understand the release kinetics (Table S4). In the case of SA and SA/LNPs-40 beads, the pseudo-second order model yielded a more accurate prediction with *R*^2^ > 0.99 than that of the pseudo-first-order model (*R*^2^ ~ 0.91). The calculated *q_e_* 48.5 mg/g highly matches with the experimental value of 48.6 mg/g. The observed pseudo-second-order kinetics suggests that the adsorption is of a chemical process with active forces between the MB and SA/LNPs-40 beads. The plot of *q_t_* vs. *t*^1/2^ is highlighted in Figure 8d. The *K_p_* estimated based on the slope of the each fitted straight-line and the *R*^2^ of SA/LNPs-40 for the step 1, step 2 and step 3 is 2.637, 0.647 and 0.170 mg/g min^1/2^ and 0.997, 0.986 and 0.871, respectively. The intercepts are not zero (Figure 8d), suggesting the occurrence of boundary layer diffusion. It further implies that intra-particle diffusion is not the only speed-control step during the MB adsorption, but it is more complicated [10,56].

#### 3.3.3. Effect of Adsorbents Dosage

**Figure 9:**
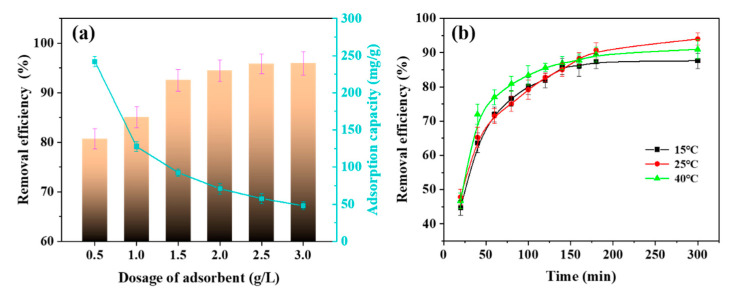
(**a**) The removal efficiency and adsorption capacity with different dosage of adsorbent (0.5–3.0 g/L SA/LNPs-40, 50 mL 150 mg/L MB, 150 rpm, 25 °C, 3 h). (**b**) Removal efficiency of different temperature (2.0 g/L SA/LNPs-40, 50 mL 150 mg/L MB, 150 rpm, 15, 25 and 40 °C, 6 h).

It could be seen from Figure 9a that the average capacity of MB adsorption decreases with increases in the adsorbent amount, while the removal rate surges and approaches the complete removal. The rise in the removal efficiency could be attributed to the presence of additional adsorption sites and the contact area provided by the higher bead content [63]. However, adsorption capacity decreases with increases of bead dosage, suggesting the generation of substantial unsaturated adsorption sites in the beads [64].

#### 3.3.4. Effect of Temperature

Figure 9b shows the effect of temperature on the removal efficiency. The removal rate is high at 2 °C, with a rate of 94.6%, followed by 91.0 and 87.6% at 40 °C and 15 °C, respectively. At the lower temperature of 15 °C, the kinetic energy of MB molecules appears to be insufficient, and thus, the rate of diffusion from the aqueous solution to the surface of the composite beads is slow. However, with the temperature rise to 40 °C, the movement rate of MB increases, and consequently, it would be easy for the MB molecules to reach the surface of the SA/LNPs beads. Although the number of MB molecules reaching the adsorption site increases, high temperature reduces forces, e.g., hydrogen bonds, between the adsorbent adsorption sites and MB molecules. Due to increases in the kinetic energy, MB molecules that are adsorbed on to the surface of the beads are desorbed, resulting in the observed decrease in the overall adsorption efficiency [61].

#### 3.3.5. Effect of Initial Concentration of MB

**Figure 10:**
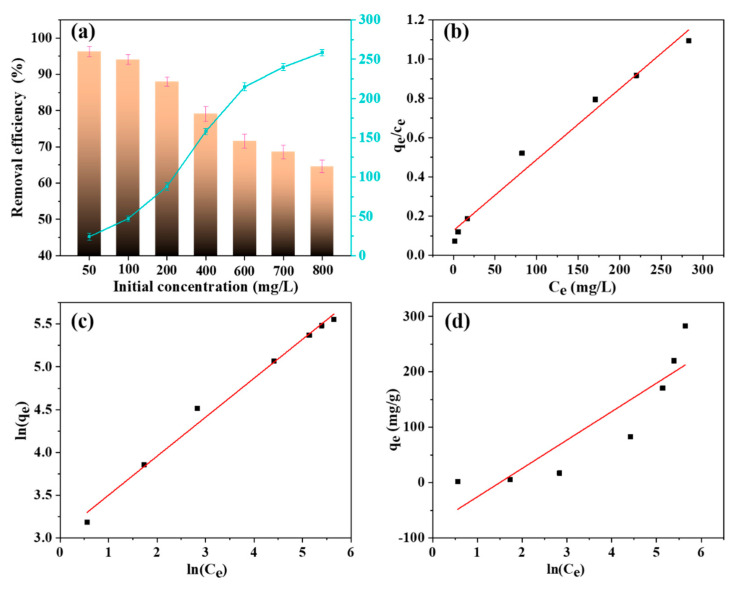
(**a**) The removal efficiency and adsorption capacity of SA/LNPs beads at different initial concentrations. (**b**) The Langmuir, (**c**) Freundlich and (**d**) Temkin model isotherms for the adsorption of MB onto the SA/LNPs-40 beads (2.0 g/L SA/LNPs-40, 50 mL 50–800 mg/L MB, 150 rpm, 25 °C, 3 h).

The effects of initial MB concentrations on the adsorption process under fixed amounts of SA/LNPs beads are studied, and corresponding plots are shown in Figure 10a. It indicates that the adsorption capacity of the SA/LNPs beads increases with the MB concentration. As the MB concentration upsurges from 50 mg/L to 800 mg/L, the adsorption quality of MB by the SA/LNPs-40 beads increases from 23.7 mg/g to 258.5 mg/g, indicating that the adsorption capacity is concentration-dependent, which could be due to higher contact probability and stronger forces between the SA/LNPs and MB molecules [65]. In order to study the adsorption process, three isotherm models, i.e., Langmuir, Freundlich and Temkin, have been tested. The model fitting curves and associated parameters are shown in Figure 10b–d and Table S5. The Langmuir (*R*^2^ ~ 0.978) and Freundlich isotherm models (*R*^2^ ~ 0.988) yield better fitting than the Temkin (*R*^2^ ~ 0.764). The Freundlich model with *n* >1 illustrates that highly favorable adsorption with strong intensity. Similarly, as per the Langmuir fitting, the maximum adsorption capacity (*q_m_*) 276.1 mg/g of MB is quite similar to the experimental observation of 258.5 mg/g. In addition, the *R_L_* value between 0 and 1 implies that the adsorption of MB onto the SA/LNPs beads is highly favorable [66,67].

Overall, SA/LNPs beads are promising, eco-friendly and effective adsorbents of MB and could as well be applicable to other dyes. The Table 2 compares the adsorption capacities of MB by some lignocellulosic-based materials, and SA/LNPs beads are comparable to cellulose nanocrystal-alginate hydrogel beads. The outcome could be easily translated to other biowastes, for example, paper industry wastes toward generating value-addition of lignin.

## 4. Conclusions

In this study, lignin nanoparticles (LNPs) have been prepared from kraft lignin using the alkaline deep eutectic solvent (DES) system without chemical modification. The obtained LNPs are spherical, with a size range of 123.6 to 140.7 nm. These LNPs are then fabricated into nanocomposite gel beads using a sodium alginate (SA) matrix to remove methylene blue (MB) from the aqueous solution. Adsorption studies reveal strong electrostatic interactions, hydrogen bonding and π-interactions between MB molecules (cationic group) and SA/LNPs (anionic functional groups), resulting in the enhanced adsorption of MB (258.5 mg/g). The outcome paves the way for further studies on the design and development of sustainable nano-lignin-based biomaterials for large-scale and industrial applications, e.g., wastewater-purification systems.

## Figures and Tables

**Table 1:** Chemical composition of the SA/LNPs composite gel beads.

Sample	H_2_O (mL)	*m*_SA_ (g)	*m*_LNPs_ (g)	*m*_LNPs_/(m_LNPs_ + m_SA_)
SA	100	1	0	0
SA/LNPs-10	100	0.9	0.1	10%
SA/LNPs-20	100	0.8	0.2	20%
SA/LNPs-30	100	0.7	0.3	30%
SA/LNPs-40	100	0.6	0.4	40%

**Table 2:** Adsorption capacities of MB by lignocellulosic-based materials reported in literatures.

Adsorbent	*q_max_* (mg/g)	Reference
Chitosan/nano-lignin composite	74	[10]
Activated lignin-chitosan extruded blends	36	[43]
Lignin-silica hybrid composites	60	[44]
Cellulose nanocrystal-alginate hydrogel beads	256	[67]
SA/LNPs beads	258.5	This study

## Data Availability

The data presented in this study are available in this article.

## Supplementary Materials

The following are available online at https://www.mdpi.com/article/10.3390/nano12010176/s1: Figure S1: Proposed mechanism of MB interactions between the dye and the LNPs; Figure S2: (a) Digital images of LNPs suspension with different concentrations and (b) average particle-size distribution of LNPs at different concentrations of aqueous solution; Figure S3: Mechanisms of cross-linking of LNPs and SA; Table S1: The chemical composition of lignin; Table S2: The information related to MB dye; Table S3: TGA results of SA/LNPs composite beads; Table S4: Parameters of various adsorption kinetic models; Table S5: Isotherm model parameters for adsorption.
