# Supplementary material for: Lignin Nanoparticles and Alginate Gel Beads: Preparation, Characterization and Removal of Methylene Blue
Source: Nanomaterials (Basel). 2022 Jan 5;12(1):176. doi: 10.3390/nano12010176 (PMC8746983; doi:10.3390/nano12010176)
Supplement: Supplementary file 1 [file nanomaterials-12-00176-s001.zip › nanomaterials-1492442-supplementary.pdf]

Supporting information

Lignin Nanoparticles and Alginate Gel Beads: Preparation, Characterization and Removal of Methylene Blue

Tong Luo [1], Yanping Hao [1], Chao Wang [1,*], Weikun Jiang [1], Xingxiang Ji [1], Guihua Yang [1], Jiachuan Chen [1], Srinivas Janaswamy [2,*] and Gaojin Lyu [1,*]

1 State Key Laboratory of Biobased Material and Green Papermaking, Qilu University of Technology, Shandong Academy of Sciences, Jinan 250353, China; luotongiant@gmail.com (T.L.); yanpingh2021@gmail.com (Y.H.); weikun0709@126.com (W.J.); jxx@qlu.edu.cn (X.J.); ygh@qlu.edu.cn (G.Y.); chenjc@qlu.edu.cn (J.C.)
2 Department of Dairy and Food Science, South Dakota State University, Brookings, SD 57007, USA
* Correspondence: chaowang@qlu.edu.cn (C.W.); Srinivas.Janaswamy@sdstate.edu (S.J.); gaojinlv@qlu.edu.cn (G.L.); Tel.: +86-0531-8963-1681 (C.W.); +86-0531-8963-1681 (G.L.)

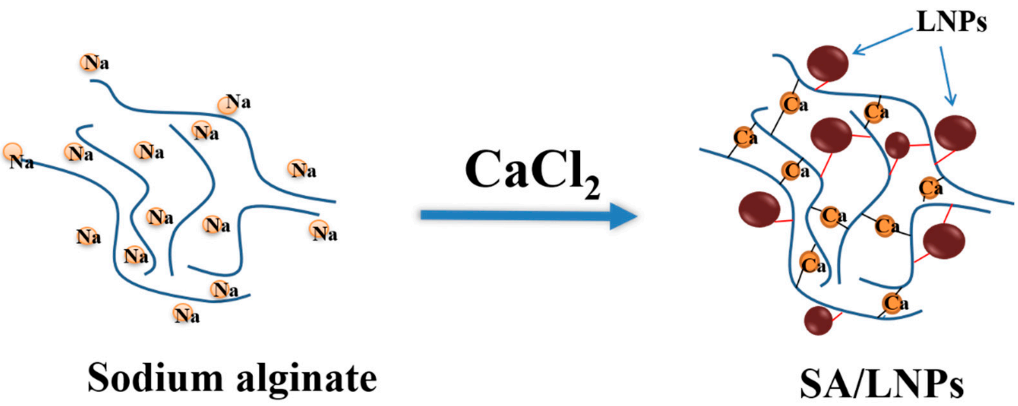

Figure S1. Mechanisms of cross-linking of LNPs and SA.

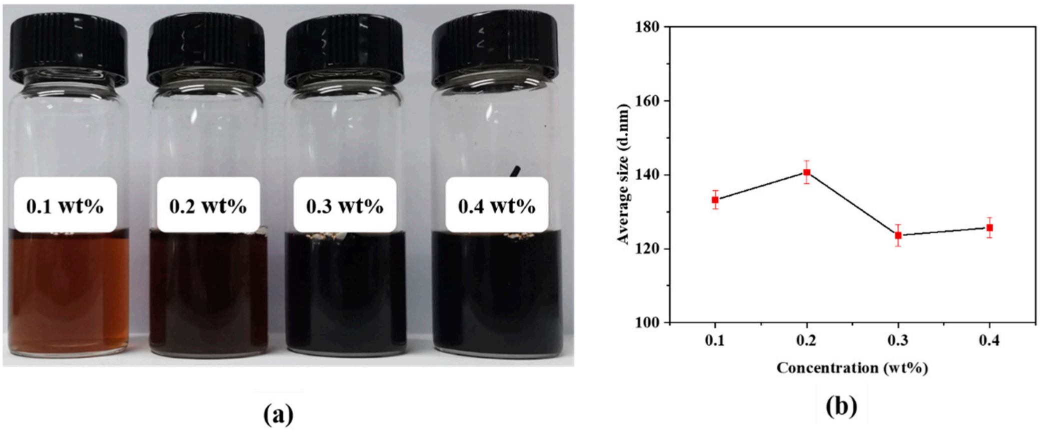

Figure S2. (**a**) Digital image of LNPs suspension with different concentrations and (**b**) average particle size distribution of LNPs at different concentrations of aqueous solution.

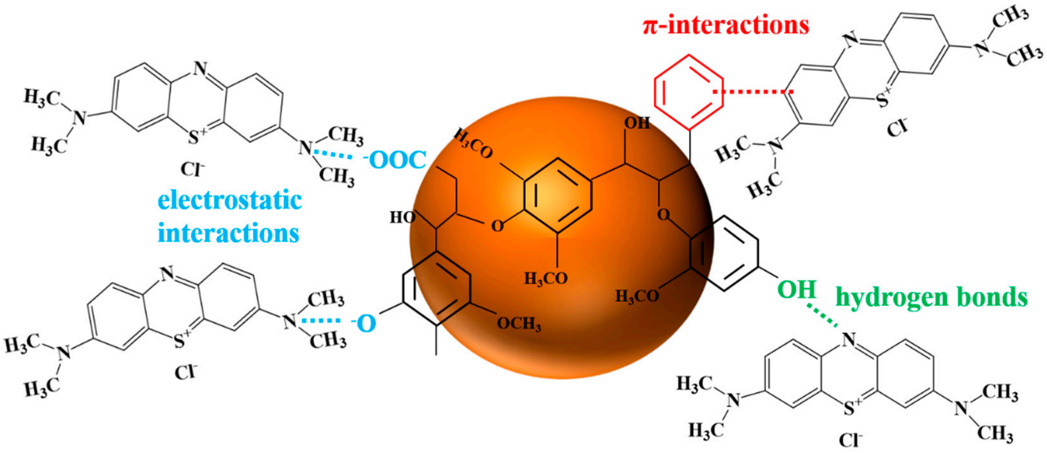

Figure S3. Proposed mechanism of MB interactions between the dye and the LNPs.

Table S1. The chemical composition of lignin.

Lignin	Acid insoluble lignin	Acid soluble lignin	Sugar	Ash
Kraft lignin	83.6%	5.8%	1.1%	7.4%

Table S2. The information related to MB dye.

IUPAC name	Maximum absorption wavelength	Formula	Molar mass	Molecular structure
3,7-Bis(dimethylamino) phenazathionium chloride	664 nm	$C_{16}H_{18}N_3SCl$	319.85 g/mol	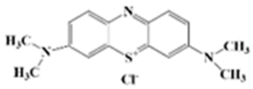

Table S3. TGA results of SA/LNPs composite beads.

Sample	T_i (˚C)	$^1T_{max}$ (˚C)	Maximum rate1 (wt%/°C)	$^2T_{max}$ (˚C)	Maximum rate2 (wt%/°C)	$W_{residue}$ (wt%)

SA powder	237.1	259.3	1.59	/	/	33.7
SA beads	225.3	233.7	0.47	299.4	0.36	37.1
SA/LNPs-10	239.7	247.8	0.33	299.7	0.34	38.1
SA/LNPs-20	235.4	252.5	0.39	304.5	0.34	43.3
SA/LNPs-30	230.2	245.6	0.29	301.8	0.30	45.2
SA/LNPs-40	239.2	258.6	0.23	300.6	0.27	48.5

Table S4. Parameters of various adsorption kinetic models.

**Kinetic model**	**Parameters**	**SA/LNPs-40**	**SA**
Pseudo-first-order model	q_e^a *(mg/g)*	48.6	44.9
	q_e^b *(mg/g)*	31.5	26.8
	k_1	0.0296	0.0256
	R^2	0.914	0.953
Pseudo-second-order model	q_e^b *(mg/g)*	48.5	44.6
	k_2	0.0055	0.0034
	R^2	0.997	0.991
Intraparticle diffusion Step 1	$k_{p1}(mg/g\ min^{1/2})$	2.637	2.037
	C_1	25.258	21.935
	R_1^2	0.985	0.927
Intraparticle diffusion Step 2	$k_{p2}(mg/g\ min^{1/2})$	0.647	0.210
	C_2	40.499	41.082
	R_2^2	0.986	0.835
Intraparticle diffusion Step 3	$k_{p3}(mg/g\ min^{1/2})$	0.170	/
	C_3	45.758	/
	R_3^2	0.871	/

[a] Experiments results, [b] Calculated results.

Table S5. Isotherm model parameters for adsorption.

**Isotherm**	**Parameters**	**SA/LNPs-40**
Langmuir	q_m *(mg/g)*	276.1
	b (L/mg)	0.287
	R^2	0.978
Freundlich	$K_F\ (mg^{1-1/n}\ L^{1/n}/g)$	21.01

	n	2.196
	R^2	0.988
	A (mg/L)	0.223
Temkin	B	51.296
	R^2	0.764

$$q_t = q_e(1 - \mathrm{e}^{-k_1 \mathrm{t}}) \rightarrow \ln(q_e - q_t) = \ln q_e - k_1 t \tag{S1}$$

$$q_t = \frac{k_2 q_e^2}{(1 + k_2 q_e t)} t \rightarrow \frac{t}{q_t} = \frac{1}{k_2 q_e^2} + \frac{t}{q_e} \tag{S2}$$

$$q_t = k_p t^{1/2} + C \tag{S3}$$

Wherein, q_t and q_e refer the amount of adsorbed MB (mg/g) at any given time and at adsorption equilibrium, respectively. The k_1, k_2 and k_p correspond to the pseudo-first-order, pseudo-second-order and the Intra-particle diffusion model rate constants, respectively. The C is the intercept (mg/g) related to the adsorption steps signifying boundary layer.

Langmuir isotherm:

$$q_e = q_{max}(\frac{bC_e}{1 + \mathrm{b}C_e}) \rightarrow \frac{q_e}{c_e} = \frac{C_e}{q_m} + \frac{1}{bq_m} \tag{S4}$$

Freundlich isotherm:

$$q_e = K_F C_e^{1/n} \rightarrow \ln q_e = \ln K_F + \frac{1}{n} \ln C_e \tag{S5}$$

Temkin isotherm:

$$q_e = B \ln A + B \ln c_e \tag{S6}$$

Herein, q_{max} (mg/g) and b (dm^3/mg) refer the constants of Langmuir isotherm; K_F (mg/g (dm^3/mg)1/n) is the adsorption capacity and $1/n$ is related to the adsorption intensity; A (mg/L) the equilibrium constant of the Temkin isotherm that is associated with binding energy and B the constant of the Temkin isotherm is related to adsorption heat.

$$R_L = \frac{1}{(1 + bC_0)} \tag{S7}$$

R_L represents the shape of isotherm with the following meanings, i.e. R_L=0 (irreversible); R_L>1 (unfavorable); R_L =1 (linear); 0<R_L<1 (favorable).
